# A Commission on Pellagra

**Published:** 1910-02-05

**Authors:** 


### A Commission on Pellagra.

While the trans-Atlantic medical journals are filled regularly with the most alarming accounts of the ravages which pellagra is causing among the population of the Southern and Western States, but little practical progress seems to have been made towards the elucidation of the ætiology of the disease. It is evident that no efforts at prophylaxis are likely to avail much until the present obscurity is dispelled: thus it is, for example, of little use to withhold maize from a population whose staple food it is, if the scourge has, in fact, nothing whatever to do with the consumption of this cereal. The *Journal of Tropical Medicine and Hygiene* announces the names of a very strong Commission which has been formed over here for the investigation of the disease. This Commission proposes to send out Dr. L. W. Sambon and, if possible, others, to some pellagrous district—most likely, it may be presumed, in Italy—to work out there the problems connected with the spread of this grave endemic. Dr. Sambon is a well-known opponent of the infected-maize theory of pellagra, and has advanced a series of reasons on which he bases the idea that some protozoal organism is the *materies morbi*. As a working hypothesis he intends to examine the relation of the various midges or sand-flies to the distribution of cases: a study of their topographical, geographical, and seasonal distribution has shown that there is a marked correspondence to that of pellagra. Whether this correspondence has been observed also in those parts of the United States where pellagra is prevalent is not stated.

Now it is not altogether a bad thing to enter upon a difficult research with a definite working theory to be tested and applied, though the disadvantages of prejudging what is entirely an open question are, of course, obvious and undeniable. If Dr. Sambon can prove that a midge or any other suctorial insect plays a constant part in the dissemination of pellagra his protozoal theory will receive strong support, and he will be very materially aided in his efforts to determine and describe the parasite itself. In such a case the vegetable-fungus theory will probably lapse into oblivion. But, though we do not object to Dr. Sambon holding strong views upon what he expects to find, he must be very wary lest he fail to keep a perfectly open mind with regard to any other factors in the ætiology. It has so often happened that investigators who have been filled with enthusiasm for some particular solution of a problem have failed to mark the plain indications pointing to some other conclusion that we feel bound to point out such a possibility. We quite agree that he has made out a case for the testing of the protozoal theory, and we hope sincerely that some definite advance towards the control of this very serious disease may be achieved as the result of the Commission's labours. We are, therefore, the more anxious that no stone shall be left unturned in the effort to work out the causation of the condition, and that every other possible explanation shall be considered and inquired into besides the parasitic theory. The duty of overseeing the results of Dr. Sambon's labours, of checking his deductions, and confirming his hypotheses will fall, without doubt, to the very able Commission which is sending him out. That entire confidence will be felt in the scientific impartiality and eminence of this body it needs only the mention of their names to demonstrate. The Commission includes Sir T. L. Brunton, Sir P. Manson, Sir T. C. Allbutt, Sir W. Leishman, Professors Ross, Osler, Minchin, and Simpson, Signor Polenghi, Drs. Mott, Sandwith, Macleod, Low, Harford, Melandri, and Sambon, Mr. Cantlie, Fleet-Surgeon Bassett-Smith, and Messrs. Austen and Wellcome. In the hands of this very strong, if perhaps too numerous, body of experts the pellagra question can very safely be left; if the funds at its disposal are adequate, some material success may not improbably be attained.

It would be very advantageous, were it possible, for this research to be carried out with some degree of correlation with the work which is being done in America. There should be, and we trust the Commission will take care that there is, no avoidable overlapping in the work. Results which have already been thoroughly tested in one hemisphere should be communicated without delay to workers in the other, so that there may be as little duplication of fruitless labour as possible. To this end an organised effort should be made to co-ordinate the various researches. A bureau similar to that of the Sleeping Sickness Bureau is perhaps hardly feasible for financial reasons. But it would be a great pity if any opportunity were neglected for making the attack on the pellagra problem a combined one of all the forces which science can put into the field.

